# Supplementary material for: Do pupillary responses during authentic slot machine use reflect arousal or screen luminance fluctuations? A proof-of-concept study
Source: PLoS One. 2022 Jul 25;17(7):e0272070. doi: 10.1371/journal.pone.0272070 (PMC9312385; doi:10.1371/journal.pone.0272070)
Supplement: S1 Text — (DOCX) [file pone.0272070.s001.docx]

**Slot machines**

Buffalo Spirit and Ice Empress are multi-line slot machines. For Buffalo Spirit, we fixed the betting strategy to the ‘maxi-min’ setting that is common among regular slot machine gamblers [1,2], wagering 1 credit (the lowest bet) on the maximum number (40) of paylines, and thus betting $0.40 per spin. This betting configuration is seen in past work to maximize the rate of reinforcement. The game had a hold setting of 11% (i.e., at infinite spins, the game keeps $0.11 from every dollar wagered). For Ice Empress, participants were allowed to vary their bets and stop and cash out whenever they wanted, wagering 1 to 5 credits on each of the 40 paylines.

**Image recognition algorithm**

Inside the slot machine casing, we doubled the video signal sent to the main game screen using an HDMI splitter (OREI, Skokie, IL), and passed a duplicate signal (using AV.IO Video Grabber; Epiphan, Palo Alto, CA) to a video capture computer, running Debut 3.01 (NCH, Greenwood Village, CO). Using an image recognition program we developed in Python 2.7 using OpenCV (Intel, Santa Clara, CA), the video files were processed to create an output file that included the ‘trial by trial’ spin outcomes, the start and end times of each spin and audiovisual feedback phase, and the credits held prior to each spin (hereafter “credit balance”).

We modified this software to also capture screen luminance. Here, frames were captured at an average rate of 53.43 frames per second (SD = .14) for Buffalo Spirit videos (n = 53), and an average rate of 48.26 frames per second (SD = 10.19) for Ice Empress videos (n = 31). Framerate was influenced by video resolution, which varied between the two types of slot machines, and within Ice Empress. However, we expect our aggregated data to overcome any systematic biases due to variable framerate (e.g., missing frames at a given time) because we had obtained so many data points.

**Results**

**Descriptive statistics**

The median participant in this experiment was exposed to a large number of loss outcomes, a smaller number of wins and losses-disguised-as-wins, and very few (if any) free spin bonus features (S1 Table). Fourteen of 53 participants did not experience a bonus feature, and there were 58 bonus features in total. Note that pupil responses could not be obtained for all outcomes (S1 Table).

**Discussion**

In addition to the pupil dilation effects at T1, we observed significant decreases in pupil diameter on win and bonus feature outcomes, relative to losses, at the end of audiovisual feedback (the T2 epoch). No pupillary changes were observed in this epoch for losses-disguised-as-wins, which lasted for a shorter duration than the (full) wins. There are at least two possible explanations for the pupil constriction effect at T2. The first is that these are genuine changes in autonomic functioning that reflect *dis*engagement from the game. Because bonus features last much longer and require no active continuation of play, it is possible that participants’ interest wanes over these extended events. This could explain why the offset of outcome feedback on bonus features was accompanied by decreased pupil diameter (Fig 2B). Another possibility is that the apparent T2 decreases arise from the chosen baseline period. Bonus features are unique in being signaled by a sequential reveal of the three over-sized symbols (see Fig 3), also accompanied by an unique audiovisual flourish (see [3]). By using the final 200 ms of the reel spin, baseline pupillary dilation may already have been amplified by the bonus feature symbols (Fig 2B), and thus the later decrease below baseline following the offset of audiovisual feedback at T2 may simply reflect physiological recovery. Based on this reasoning, we also note that the true effect size for the pupillary increases for the onset of bonus feature feedback (at T1) may be underestimated in our analysis.

**Additional Limitations**

Several additional limitations should be noted. Although the pupillary response has a superior temporal resolution compared to some other widely-used physiological parameters such as skin conductance, the losses-disguised-as-wins in our slot machine task were almost always shorter than the 2,500 ms response epoch defined for T1. As a result, the T1 and T2 epochs partly overlap for losses-disguised-as-wins. In future studies, researchers may consider employing a pupil deconvolution analysis (e.g., [4]).

Pupil diameter is governed by both the sympathetic and parasympathetic nervous systems, and the complex interactions of these systems may not be easily separable (e.g., [5]). While the relatively high temporal resolution of pupillary methods offers some promise for measuring autonomic responses to genuine slot machine events, slot machine pupillometry experiments would benefit from careful consideration of other factors that could impact the pupil light reflex besides luminance, such as different colors, visual flicker, or the multi-modal impact of win-concurrent sound feedback.

**Correcting for pupillary foreshortening was not possible in Experiment 1**

The Pupil Core system employed in Experiment 2 uses a three dimensional model of pupil size that is more robust against foreshortening error [6]. Experiment 1 used SMI’s BeGaze analysis software, which provided less robust estimates of pupil diameter. Although participants in that study spent the overwhelming majority of the task staring straight ahead at the slot machine screen [7], results in Experiment 1 could have been impacted by participants’ gaze positions during a particular event relative to the position of each eye tracking camera (i.e., foreshortening errors).

Using available point-of-regard data from Experiment 1, we examined the correlation between uncorrected pupil diameter measurements (in millimeters), and measurements which had been corrected for pupillary foreshortening using a validated linear regression procedure [8,9]. The individual correlations between corrected and uncorrected pupil diameters are presented in S1 Fig. In general, there was a high correlation between corrected and uncorrected pupil diameters. In the median participant, corrected pupil diameter explained a majority (*r*^2^ = 0.73) of the variation in uncorrected pupil diameter, indicating that approximately 27% of variance in pupil diameter was explained by the foreshortening correction model. For comparison, in Brisson et al (2013), 20% of the variance in uncorrected pupil diameter measurements could be explained by the foreshortening-correction regression model.

Crucially, the abrupt closure of SMI in 2017 prevented us from accessing the raw point-of-regard data (prior to our mapping of the data using SMI’s proprietary Semantic Gaze Mapping software), and this impaired the potential utility of our ‘corrected’ pupil diameter measurements. For these data, Semantic Gaze Mapping entailed manually identifying each point-of-regard using a reference image. The borders on this reference image are limited to the size of the slot machine screen, meaning that data pertaining to the visual periphery were lost. As such, our correction models lacked the necessary point-of-regard data needed to fix the most serious instances of pupillary foreshortening. These regression-based foreshortening corrections thus created potentially-misleading measurements for our data. Our analyses are therefore reported without correcting for pupillary foreshortening.

**References**

[1] Livingstone C, Woolley R, Zazryn T, Bakacs L, Shami R. The Relevance and Role of Gaming Machine Games and Game Features on the Play of Problem Gamblers REPORT Prepared for: Independent Gambling Authority South Australia EGM games and game features Report. 2008. https://doi.org/10.13140/RG.2.1.3070.2249.

[2] Templeton JA, Dixon MJ, Harrigan KA, Fugelsang JA. Upping the reinforcement rate by playing the maximum lines in multi-line slot machine play. Journal of Gambling Studies 2015;31:949–64. https://doi.org/10.1007/s10899-014-9446-5.

[3] Dixon MJ, Templeton J, Collins K, Wojtowicz L, Harrigan K, Fugelsang J, et al. Cross-modal attention in the “reel” world: Visual and auditory influences on near-misses in multi-line slot machine play. The handbook of attention, Cambridge, MA: MIT Press; 2015, p. 678.

[4] Wierda SM, van Rijn H, Taatgen NA, Martens S. Pupil dilation deconvolution reveals the dynamics of attention at high temporal resolution. Proceedings of the National Academy of Sciences of the United States of America 2012;109:8456–60. https://doi.org/10.1073/pnas.1201858109.

[5] Steinhauer SR, Siegle GJ, Condray R, Pless M. Sympathetic and parasympathetic innervation of pupillary dilation during sustained processing. International Journal of Psychophysiology 2004;52:77–86. https://doi.org/10.1016/j.ijpsycho.2003.12.005.

[6] Petersch B, Dierkes K. Gaze-angle dependency of pupil-size measurements in head-mounted eye tracking. Behavior Research Methods 2021. https://doi.org/10.3758/s13428-021-01657-8.

[7] Murch WS, Limbrick-Oldfield EH, Ferrari MA, MacDonald KI, Fooken J, Cherkasova MV, et al. Zoned In or Zoned Out? Investigating Immersion in Slot Machine Gambling using Mobile Eye Tracking. Addiction 2020;115:1127–38. https://doi.org/10.1111/add.14899.

[8] Brisson J, Mainville M, Mailloux D, Beaulieu C, Serres J, Sirois S. Pupil diameter measurement errors as a function of gaze direction in corneal reflection eyetrackers. Behavior Research Methods 2013;45:1322–31. https://doi.org/10.3758/s13428-013-0327-0.

[9] Martin JT, Whittaker AH, Johnston SJ. Component processes in free-viewing visual search: Insights from fixation-aligned pupillary response averaging. Journal of Vision 2020;20:1–24. https://doi.org/10.1167/JOV.20.7.5.
