# Supplementary material for: Do pupillary responses during authentic slot machine use reflect arousal or screen luminance fluctuations? A proof-of-concept study
Source: PLoS One. 2022 Jul 25;17(7):e0272070. doi: 10.1371/journal.pone.0272070 (PMC9312385; doi:10.1371/journal.pone.0272070)
Supplement: S1 Table — Loss outcomes do not involve any audiovisual feedback and thus we could not specify a T2 event (that was distinct from T1). Bonuses include all free spins during the Audiovisuals phase. LDW = Loss-Disguised-as-a-Win. (DOCX) [file pone.0272070.s002.docx]

| Outcome Type | Events per Participant median (range) | Duration of Spin Reel median (range) | Duration of Audiovisuals median (range) | Duration of Spin Initiation Latency median (range) | Total Events | Pupil Responses Analyzed [T1, T2] |
| --- | --- | --- | --- | --- | --- | --- |
| Loss | 140 (95 – 190) | 3.20 (1.03 – 12.10) | – | 1.17 s (0.00 – 45.77) | 7451 | [3912, – ] |
| Win | 17 (8 – 28) | 3.20 (1.03 – 14.67) | 2.36 s (0.05 – 72.73) | 2.04 s (0.00 – 18.58) | 876 | [460, 452] |
| LDW | 18 (10 – 33) | 3.17 (1.13 – 11.45) | 0.47 s (0.18 – 15.60) | 1.80 s (0.00 – 63.18) | 1016 | [508, 502] |
| Bonus | 1 (0 – 4) | 9.20 (1.37 – 11.87) | 75.23 s (45.52 – 196.02) | 2.57 s (0.00 – 13.18) | 58 | [28, 29] |
