# Supplementary figures and images for: Do pupillary responses during authentic slot machine use reflect arousal or screen luminance fluctuations? A proof-of-concept study
Source: PLoS One. 2022 Jul 25;17(7):e0272070. doi: 10.1371/journal.pone.0272070 (PMC9312385; doi:10.1371/journal.pone.0272070)

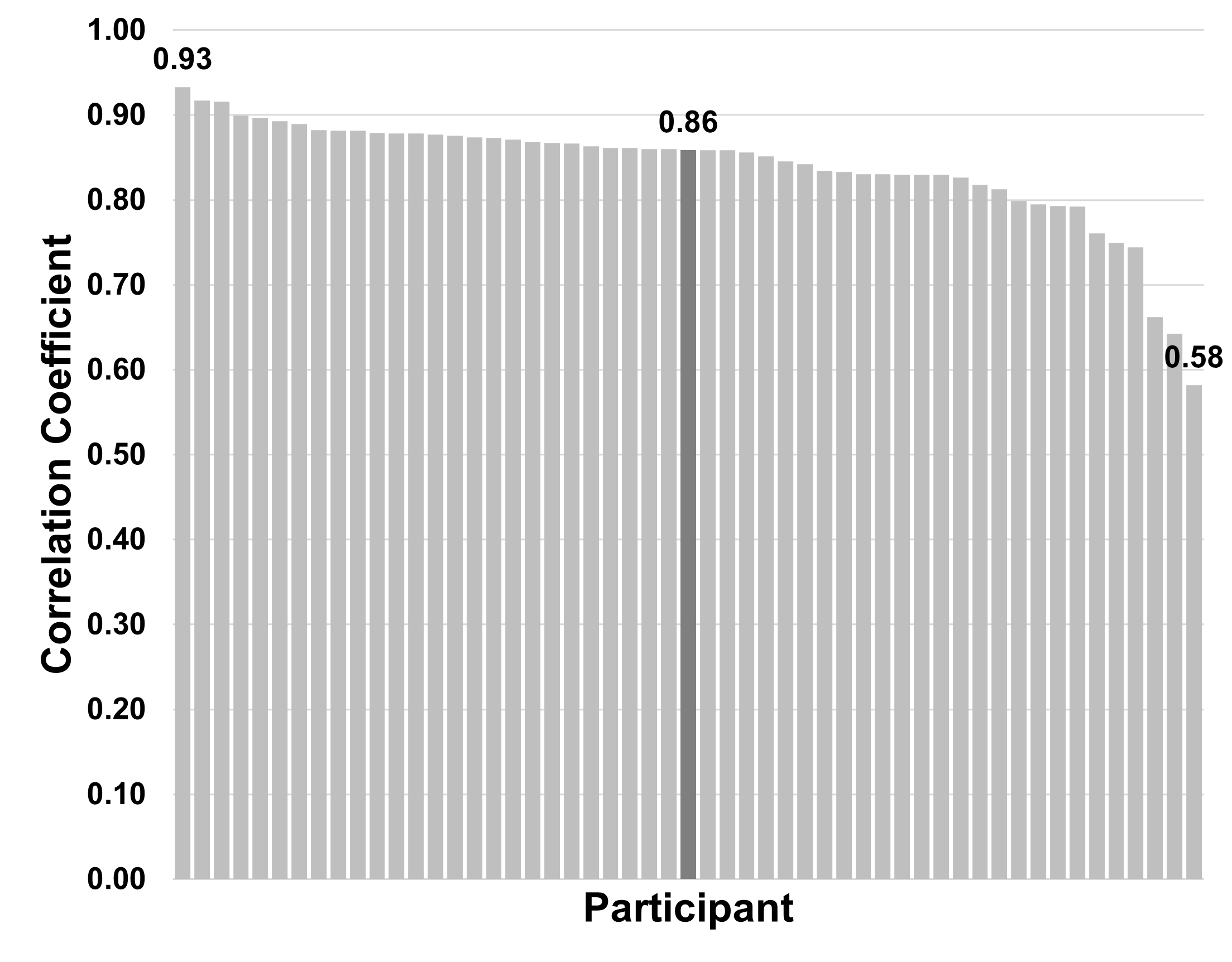

Supplement: S1 Fig — Within-subjects correlations for each of the 53 participants in Experiment 1 are shown. Data labels depict the maximum, median, and minimum correlations. (TIF) [file pone.0272070.s003.tif]
